# Effects of dietary supplement with a Chinese herbal mixture on growth performance, antioxidant capacity, and gut microbiota in weaned pigs

**DOI:** 10.3389/fvets.2022.971647

**Published:** 2022-08-22

**Authors:** Qinglei Xu, Meng Cheng, Rong Jiang, Xianle Zhao, Jianjin Zhu, Mingzheng Liu, Xiaohuan Chao, Chunlei Zhang, Bo Zhou

**Affiliations:** ^1^College of Animal Science and Technology, Nanjing Agricultural University, Nanjing, China; ^2^Wuxi Sanzhi Bio-Tech Co., Ltd., Wuxi, China; ^3^College of Food Science and Technology, Jiangnan University, Wuxi, China

**Keywords:** weaned pigs, herbal mixture, growth performance, antioxidant capacity, gut microbiota

## Abstract

Weaning stress decreases the growth performance of piglets and is one of the main concerns of pig industries. Traditional Chinese herbal medicines have been used to reduce the adverse effects of weaning stress as both nutritional supplements and antibiotic substitutes. This study aimed to evaluate the effects of a Chinese herbal mixture (Kangtaile, which contained Paeonia lactiflora, licorice, dandelion, and tea polyphenols) on the growth performances, immune response, antioxidant capacity, and intestinal microbiota of weaned pigs. A total of 400 weaned pigs [Duroc × (Landrace × Yorkshire)] were randomly allocated into one of four treatments: the CON group, fed with basic diet; the HM1 group, fed with basal diet supplemented with 0.5 g herbal mixture/kg diet; the HM2 group, fed with basal diet supplemented with 1.0 g herbal mixture/kg diet; or the HM3 group, fed with basal diet supplemented with 1.5 g herbal mixture/kg diet. The results revealed that dietary supplementation with the herbal mixture for 28 days improved average daily gain and feed conversion ratio, while decreased the diarrhea rate of weaned pigs. Moreover, dietary supple-mentation with the herbal mixture improved the antioxidant capacity through increasing the activity of catalase (CAT) and the total antioxidant capacity (T-AOC) level, while decreasing the concentration of malondialdehyde (MDA) in the serum. Pigs supplemented with herbal mixture presented an increased serum immunoglobulin (Ig)M level on day 14 compared with control pigs. The herbal mixture altered the composition of intestinal microbiota by influencing the relative abundances of Firmicutes and Bacteroidetes at the phylum level. The relative abundances of the Firmicutes and Bacteroidetes were significantly related to the body weight gain of pigs. In conclusion, supplementation of herbal mixture to the diet improved growth performance, immunity, and antioxidant capacity and modified the composition of intestinal microbiota in weaning pigs. This study provided new insights into the nutritional regulation effects of the herbal mixtures on weaned pigs.

## Introduction

Weaning stress often leads to diarrhea, respiratory diseases, and loss of weight gain in weaned pigs (1). Antibiotic additives were thus used to reduce weaning stress in the feed of weaned pigs (2). However, the abuse of antibiotic drugs led to antibiotic residues in animal derived foods, while antibiotic-resistant bacteria were transmitted to humans through the food chain, which led to serious health hazards for humans (3). Therefore, with the ban on antibiotics as feed additives, it is urgent to identify the safe alternatives to antibiotics to enhance the protection of both animal derived foods and human health.

In livestock production, Chinese herbal medicines have been considered as safe feed additives to substitute antibiotics in animal feed (4, 5). Because Chinese herbal medicines are natural products, they contain a variety of beneficial ingredients that maintain the overall health of animals and prevent diseases in animals (6). A previous study found that Paeoniflorin is an effective active ingredient in Paeoniae Radix Alba (PRA) (dried root of *Paeonia lactiflora Pall*) and had anti-inflammatory and immune regulation functions (7). Dietary supplementation with peony pollen improved the growth performance, digestive capacity, catalase, and total antioxidant capacity and reduced malondialdehyde level in carps (8). Licorice has anti-inflammatory and antiviral properties, and its main effective active ingredient is glycyrrhizin (9). Previous studies have revealed that the supplementation of seaweed and licorice increased the concentration of IgA and decreased the mRNA expression level of tumor necrosis factor-α in the saliva of pigs (10). The main active ingredients of dandelion are sesquiterpene lactone and phenylpropanoid polysaccharide, which have anti-inflammatory and immune regulating effects (11). A combination of 5 g/kg garlic and 50 g/kg dandelion improved fattening pig growth and carcass quality (12). Tea polyphenols (TP) are the main active ingredients in tea and have the functions of antioxidants, obesity reduction, and disease risk reduction (13). In addition, a previous study indicated that dietary supplementation of 0.2% TP increased the relative abundance of *Lactobacillus*, also decreased the abundance of *Bacteroidacease* and the total bacteria count in pigs (14).

However, previous studies have not mentioned the use of combinations of herbs to reduce the negative effects of weaning stress in pigs. Furthermore, the mechanism of herbal combination formulation to improve the health and physiological condition of pigs has not been clarified. In general, multiple active ingredients in herbal mixture composed of several herbs have synergistic effects, and their biological efficiency is greater than that of a single herbal extract (15, 16). Furthermore, the factors influencing the production performance of weaning pigs are complex, so the effect of a single herbal medicine is often limited in the treatment of these complicated problems caused by weaning stress (17). Previous studies have revealed that the synergistic effects of herbal mixture are achieved by applying a pair of herbs with similar therapeutic functions (18). For example, the coexisting constituents in a herbal mixture promoted the intestinal absorption of active constituents by improving solubility and increasing the membrane permeability of enterocytes (19). The synergistic effect of herbal mixture also indicated that the combined preparation reduces the toxicity of one of the herbs through the selective metabolism of the main active components, which achieved the therapeutic effect through metabolic detoxification (20). Shaoyao-Gancao Decoction (SGD), a classical formulated Chinese herbal medicine, consists of Paeoniae Radix Alba and licorice (21), which has pharmacological functions of antioxidation and immune regulation (22, 23). Similarly, the main ingredients of 24 flavor tea are dandelion and licorice, which have protective effects on immune rejection during organ transplantation (24). However, the combined effects of these herbal mixture (R. paeoniae alba, Licorice, Dandelion and Tea polyphenols) have not been investigated in weaned pigs. Therefore, this study aimed to investigate the effects of herbal mixture on the growth performance, immunity, antioxidant capacity, and intestinal microorganisms of weaned pigs. The present study provides experimental data for supplementation of the herbal mixture as an alternative to reduce post-weaning stress in pigs.

## Materials and methods

### Preparation of the herbal mixture (Kangtaile)

A type of herbal mixture called Kangtaile was prepared by Wuxi Sanzhi Biotechnology Co., Ltd (Wuxi, China). The herbal mixture is a mixed combination of five commonly used herbal medicines with different proportions including: Paeoniae Radix Alba (42%), licorice (28%), dandelion (28%), and tea polyphenols (2%). The ratio of Paeoniae Radix Alba and licorice was revised on the basis of the traditional Chinese medicine prescription “Shaoyao Gancao Decoction” (25). In addition, the combination of licorice and dandelion has the functions of antioxidant, anti-inflammatory and antibacterial (26). They exist simultaneously as the main components in tea drinks (27). The herbs are dried, broken and crushed into powder, as well as screened through an 800-mesh sieve before use, and then mixed by proportion to obtain herbal mixture (dark brown powder), which was stored in a sealed container at room temperature before use. Total polysaccharides, total saponins, flavonoids, and polyphenols are the main antioxidant compounds (28). The compositions of total polysaccharide and total flavonoids in herbal mixtures mainly come from Paeoniae Radix Alba, licorice and dandelion. The compositions of total saponins in herbal mixtures mainly come from licorice. The compositions of total polyphenols in herbal mixtures mainly come from Paeoniae Radix Alba, dandelion, and tea polyphenols. The principal active antioxidant components (total polysaccharide, and total saponins) of herbal mixture were determined by ultra-performance liquid chromatography (UPLC) method based on a previous study (29). In brief, the UPLC analysis was performed using a UPLC-Xevo TQ-S mass spectrometer (Waters Xevo TQ MS, Waters Corp., Milford, MA, USA). The following UPLC condition was used: the chromatographic column is a Kinetex C18 column (2.1 × 100 mm, 1.7 μm); Mobile phase A was acetonitrile and mobile phase B was 0.1% formic acid and injection volume 1 μL. A flow rate of 0.15 mL/min; column temperature: 45°C; total running time 12 min. Total polyphenols content was determined according to the Folin-Ciocalteu chromatometry method (30). Absorbance of the sample was measured with the microplate reader (Tecan, Austria GmbH, Grödig, Austria) at 760 nm. The total phenolic content was expressed as milligram of gallic acid equivalent per gram of herbal mixture. The determination of total flavonoids was performed by NaNO_2_-Al(NO_3_)_3_-NaOH colorimetric method with rutin as a standard for estimating the content of total flavonoids (31). After determination, the concentration of total polysaccharide, total saponins, total polyphenols and total flavonoids in the herbal mixture were 70.5, and 24.8, 13.0, and 26.2 mg/g, respectively.

### Experimental design, animals, and housing

This study was approved by the Animal Care and Use Committee of Nanjing Agricultural University (SYXK2017-0027, Nanjing, China). The experiments were conducted at Jiangsu Huaduo Animal Husbandry Technology Co., Ltd. (Xuzhou, China). A total of 400 post-weaned pigs [Duroc × (Landrace × Yorkshire)] (equal number of males and females) with body weight (BW) about 8.37 ± 0.11 kg and age of 28 days were blocked according to BW and sex and then randomly allocated into one of four treatments: the CON group, fed with the basal diet (Supplementary Table 1); the HM1 group, fed with the basal diet supplemented with 0.5 g herbal mixture/kg diet; the HM2 group, fed with the basal diet supplemented with 1.0 g herbal mixture/kg diet; or the HM3 group, fed with the basal diet supplemented with 1.5 g herbal mixture/kg diet. There were 4 replicates (containing 2 male and 2 female pig pens) per treatment and 25 pigs per pen. The detailed feed formula and the nutritional levels were presented in Supplementary Table 1. All experimental pigs were raised in the same environment for 28 days. All pens were thoroughly cleaned and disinfected to reduce viral and bacterial contamination before the pigs moved in. During the trial, air-exhaust fans and hot-blast heaters were equipped to automatically control the room temperature between 21 and 25°C. The pens (3.5 × 4.5 m) were equipped with slatted plastic floors, an adjustable stainless-steel feeder, and two nipple drinkers for *ad libitum* access to feed and water.

### The growth performance of weaned pigs

The weaned pigs and feed consumed were weighed on d 0, 14, and 28 of the experiment period to calculate the growth performances: average daily gain (ADG), average daily feed intake (ADFI), and feed to gain ratio (F: G). The diarrhea rates were determined based on the diarrhea score of pigs at the end of the experiment. The feces of weaned pigs were assessed regularly from 08:00 to 09:00 every day according to the criteria: 1-solid, well-formed feces; 2-loose and shapeless feces; 3-runny feces; and 4-watery diarrhea (32). Pigs with a fecal score ≥ 3 were considered to have diarrhea; pigs with a fecal score of <3 were considered normal. Diarrhea symptoms and mortality, if any, were recorded daily for each pig during the trial. The diarrhea rate was calculated as the (number of pigs with diarrhea)/(number of pigs tested × total experiment days) × 100%.

### Complete blood count examination

A total of 64 pigs (8 male and 8 female pigs per treatment) were randomly selected to collect 5 mL blood *via* jugular venipuncture into anticoagulant (heparin) tubes on day 14 and 28 of the experimental period. The blood was then divided equally into two parts: one for complete blood count (CBC) test and the other for determination of antioxidant indicators. A total of 16 CBC indicators were measured by an automatic biochemical analyzer (Mindray BC-5000, Mindray Medical International Co. LTD, Shenzhen, China). These CBC indicators include white blood cell number (WBC), Neutrophil number (NEU), Lymphocyte number (LYM), monocyte number (MON), eosinophils number (EOS), basophils number (BAS), Red blood cell number (RBC), Hemoglobin (HGB), Erythrocyte specific Volume (HCT), Mean Erythrocyte Volume (MCV), Mean Erythrocyte Hemoglobin content (MCH), Mean Red blood cell hemoglobin concentration (MCHC), platelet count (PLT), mean platelet volume (MPV), platelet distribution width (PDW), and platelet hematocrit (PCT).

### Antioxidant capacity and immunoglobulin M determination

The serum samples (*n* = 64) were obtained by centrifuging (4,000 × *g* for 10 min) at 4°C, and stored at −80°C until analysis. Six oxidative stress indicators, such as malondialdehyde (MDA), total superoxide dismutase (SOD), glutathione (GSH), glutathione peroxidase (GSH-Px), catalase (CAT), and total antioxidant capacity (T-AOC) were determined using ELISA kits (Nanjing Jiancheng Institute of Biological Engineering, Nanjing, China) according to the manufacturer's instructions. The sensitivities of the kits are as follows: MDA: 0.1 nmol/mL; SOD: 0.1 U/mL; GSH: 10 μmol/L; GSH-Px: 0.2 mg/mL; CAT: 0.2 U/mL; T-AOC: 0.1 mmol/gprot. The intra- and inter-assay coefficients of variation were <10 and 15%, respectively. Concentration of immunoglobulin M (IgM) was determined using an ELISA kit (Nanjing Jiancheng Institute of Biological Engineering, Nanjing, China) with an assay sensitivity of 0.1 mg/ml according to the manufacturer's instruction. A microplate reader (Tecan, Austria GmbH, Grödig, Austria) was used to determine all indicators. The intra- and inter-assay of the coefficient of variation were <10 and 15%, respectively.

### Fecal sample collection, DNA extraction, and 16S rRNA gene sequencing

On day 14 and 28 of the experimental period, a total of 96 weaned pigs (6 male and 6 female pigs per treatment) were randomly selected to collect fresh feces using clean plastic bags into a 1.5 mL centrifugation tube and stored at −80°C until analysis. Genomic DNA of fecal samples was extracted by the hexadecyltrimethylammonium bromide (CTAB) method (33), and DNA purity was detected by agarose gel electrophoresis. The genomic DNA was used as the template. The V3–V4 region of the bacterial 16S rRNA gene was amplified with the specific primers (F: 5′-GTGYCAGCMGCCGCGGTAA-3′, R: 5′-GGACTACHVGGGTWTCTAAT-3′). The PCR products were confirmed with 2% agarose gel electrophoresis, purified with AMPure XT beads (Beckman Coulter Genomics, Danvers, MA, USA), and then quantified by an Invitrogen Qubit 4.0 fluorometer (Invitrogen, Thermo Fisher Scientific, Waltham, MA, USA). For library construction, the NEBNext^®^ Ultra™ DNA Library Prep Kit (Illumina, United States) was used. After the library was qualified, the library was sequenced on an Illumina next-generation sequencing platform NovaSeq 6000 to generate the 250 bp paired-end reads. The original sequencing data were spliced and filtered to obtain clean reads. Then, through Divisive Amplicon Denoising Algorithm 2 (DADA2), the sequences with an abundance <5 were filtered out to obtain the final Amplicon Sequence Variants (ASVs) (34). Each de-weighting sequence generated after DADA2 is called ASVs, which corresponds to the Operational Taxonomic Units (OTUs) representative sequence. The available data were then annotated and abundances were analyzed to reveal the species composition, and further *Alpha* and *Beta* diversity analyses were performed to explore differences in community structure.

### Statistical analysis

Data on growth performances (body weight, average daily gain, average daily feed intake, feed to gain ratio), CBC test, antioxidant indicators, and relative abundance of microbiota were analyzed using the GLIMMIX procedure of SAS® software 9.4 (SAS Institute, Inc., Cary, NC, USA). The model included dietary treatment, gender, and their interaction as fixed effects and pen as random effect. The experimental units for the growth performance parameters (average daily gain, average daily feed intake, and feed to gain ratio) and diarrhea rate were the pen, while the experimental units for the antioxidant indicators, blood hematology parameters and gut microbiota was the individual pig. A partial correlation analysis between the gut microbiota and growth performance indicators, antioxidant indicators was carried out using R statistical software. Graphs were performed using Graphpad Prism 8.0 (GraphPad Software Inc., San Diego, CA, USA). The results were expressed as the mean ± standard error of the mean (SEM). All tests were considered statistically significant at *p* < 0.05 and tendencies at 0.05 < *p* < 0.10. *P*-values were rounded to three digits after the decimal point: *p*-values < 0.001 are shown as < 0.001.

## Results

### Growth performance and diarrhea rate

The effects of the herbal mixture on the growth performance and the diarrhea rate of weaned pigs were presented in Table 1. There was no significant effect of dietary treatment, sex or treatment × sex interaction on initial body weight and body weight on day 14 (*p* > 0.05). On day 28 of the experiment period, the body weight of pigs in the HM1, HM2, and HM3 groups were greater than that of pigs in the CON group (19.17, 19.73, and 19.26 vs. 18.70 kg, *p* < 0.001). During the first period (day 1–14), dietary supplementation with herbal mixture tended to increase ADG compare to the CON group (325.52, 340.16, and 322.77 vs. 307.99 g/day, *p* = 0.064). ADFI in the HM3 group was less than that in the CON group (464.50 vs. 479.84 g/day, *p* = 0.002). Female pigs had a higher ADFI compared with male pigs (478.84 vs. 472.33 g/day, *p* = 0.018). Dietary supplementation with herbal mixture had a lower F: G compared with pigs offered the basal diets (1.48, 1.40, and 1.44 vs. 1.56, *p* = 0.004). During the second period (day 15–28), ADFI in the HM1 and HM2 groups were greater than that in the CON group (673.93 and 676.62 vs. 659.89 g/day, *p* = 0.009). Female pigs had a higher ADFI compared with male pigs (674.41 vs. 664.88 g/day, *p* = 0.007). In the male pigs, dietary supplementation with herbal mixture had a greater ADFI compared with pigs offered the basal diets (674.57, 675.10, and 664.50 vs. 645.36 g/day, *p* = 0.015). Pigs offered herbal mixture had a lower F: G compared with the pigs offered the basal diets (1.49, 1.41, and 1.49 vs. 1.56, *p* < 0.001). Male pigs had a lower F: G compared with female pigs (1.46 vs. 1.51, *p* = 0.005). In the male pigs, F: G in the HM2 group was less than that of pigs in the CON group (1.42 vs. 1.49, *p* = 0.024). In the female pigs, dietary supplementation with herbal mixture had a lower F: G compared with pigs offered the basal diets (1.51, 1.40, and 1.52 vs. 1.62, *p* = 0.024). During the whole experiment period, dietary supplementation with herbal mixture had a greater ADG compared with pigs offered the basal diets (388.99, 409.78, and 386.22 vs. 365.74 g/day, *p* = 0.002). ADFI in the HM3 group was less than that in the CON group (566.32 vs. 576.59 g/day, *p* < 0.001). Dietary supplementation with herbal mixture had a lower F: G compared with pigs offered the basal diets (1.49, 1.41, and 1.47 vs. 1.58, *p* < 0.001). During the whole 28-day experimental period, dietary supplementation with herbal mixture had a lower diarrhea rate compared with pigs offered the basal diets (4.86, 4.46, and 3.79 vs. 6.30, *p* < 0.001).

**Table 1:** Effects of herbal mixture on growth performance of weaned pigs^1^.

**Items**	**Dietary treatments** ^**2**^								**SEM^3^**	***p*** **-value**		
	**Male**				**Female**							
	**CON**	**HM1**	**HM2**	**HM3**	**CON**	**HM1**	**HM2**	**HM3**		**Treatment**	**Sex**	**Treatment × sex**
**BW (kg)**												
Day 1	8.50	8.29	8.22	8.64	8.44	8.29	8.28	8.26	0.11	0.385	0.398	0.470
Day 14	12.78	12.82	13.07	12.99	12.76	12.85	12.98	12.93	0.12	0.435	0.761	0.984
Day 28	18.82^bc^	19.22^abc^	19.71^a^	19.43^ab^	18.59^c^	19.13^abc^	19.76^a^	19.09^abc^	0.17	<0.001	0.390	0.887
**Days 1–14**												
ADG (g)	305.69^b^	324.12^ab^	345.54^a^	311.03^b^	310.30^b^	326.92^ab^	334.79^ab^	334.51^ab^	9.78	0.064	0.487	0.427
ADFI (g)	479.61^a^	480.57^a^	467.27^b^	461.86^b^	480.07^a^	483.37^a^	484.79^a^	467.14^b^	3.09	0.002	0.018	0.094
F: G	1.57^a^	1.48^ab^	1.35^c^	1.48^ab^	1.55^a^	1.48^ab^	1.44^bc^	1.40^bc^	0.03	0.004	0.831	0.083
**Days 15–28**												
ADG (g)	432.42	457.96	474.54	458.68	414.54	446.96	484.25	440.67	22.12	0.174	0.569	0.910
ADFI (g)	645.36^c^	674.57^ab^	675.10^ab^	664.50^b^	674.43^ab^	673.29^ab^	678.14^a^	671.79^ab^	3.74	0.009	0.007	0.015
F: G	1.49^bc^	1.47^bcd^	1.42^de^	1.45^cde^	1.62^a^	1.51^bc^	1.40^e^	1.52^b^	0.02	<0.001	0.005	0.024
**Days 1–28**												
ADG (g)	369.05^bc^	391.04^ab^	410.04^a^	384.86^bc^	362.42^c^	386.94^ab^	409.52^a^	387.59^ab^	7.30	0.002	0.691	0.922
ADFI (g)	575.93^ab^	577.57^a^	578.08^a^	563.18^c^	577.25^a^	578.33^a^	581.46^a^	569.46^bc^	2.09	<0.001	0.082	0.569
F: G	1.56^a^	1.48^bc^	1.41^d^	1.46^bcd^	1.59^a^	1.49^b^	1.42^cd^	1.47^bcd^	0.02	<0.001	0.278	0.915
Diarrhea rate (%)	6.38^a^	4.50^b^	4.42^bc^	3.50^c^	6.21^a^	5.21^b^	4.50^bc^	4.07^c^	0.34	<0.001	0.251	0.561

*Least-square means ± S.E.M*.

*BW, body weight; ADG, average daily gain; ADFI, average daily feed intake; F: G, feed to gain ratio*.

*^1^Data were the mean of 100 replicates per treatment*.

*^2^Dietary treatments: CON, the control group, fed with the basal diet; HM1, the herbal mixture group 1, fed with the basal diet supplemented with 0.5 g herbal mixture/kg diet; HM2, the herbal mixture group 2, fed with the basal diet supplemented with 1.0 g herbal mixture/kg diet; HM3, the herbal mixture group 3, fed with the basal diet supplemented with 1.5 g herbal mixture/kg diet*.

*^3^SEM = Standard error of the mean*.

*^a−d^ Values in the same row not sharing a common superscript mean a significant difference (p < 0.05)*.

### Serum antioxidant indicators and IgM concentration

The effects of the herbal mixture on the antioxidant capacity of weaned pigs were presented in Table 2. There were evident tendencies of dietary treatment × sex interaction effects on the level of SOD (*p* = 0.060) and GSH (*p* = 0.052) in pigs on day 14. In the male pigs, the activity of SOD in the HM1 group was greater than that in the CON group (166.68 vs. 155.14 U/mL, *p* = 0.046). In the male pigs, the activity of GSH in the HM2 and HM3 were greater than that in the CON group (64.67 vs. 58.47 μmol/L, *p* = 0.036, 64.92 vs. 58.47 μmol/L, *p* = 0.037, respectively). On day 14, male pigs had a higher activity of GSH-Px compared with female pigs (671.54 vs. 620.64 mg/mL, *p* = 0.016). On day 14, the level of T-AOC in the HM3 group was significantly greater than that in the CON group (5.73 vs. 5.62 U/mL, *p* = 0.001). The concentration of MDA in the female pigs had a tendency to decrease compare with the male pigs (2.97 vs. 3.14 nmol/mL, *p* = 0.075). Dietary supplementation with herbal mixture had a greater concentration of IgM compared with the pigs offered the basal diets (1.91, 1.91, and 1.91 vs. 1.86 mg/mL, *p* = 0.026). On day 28, the dietary supplementation with herbal mixture significantly increased the activity of CAT in weaned pigs (6.77, 6.89, and 6.80 vs. 5.97 U/mL, *p* = 0.022) compared with the CON group. The level of T-AOC in the HM groups was significantly greater than that in the CON group (4.68, 4.67, and 4.58 vs. 4.36 U/mL, *p* = 0.018). The concentration of MDA in the HM2 and HM3 group was significantly less than that in the CON group (3.12 and 2.97 vs. 3.50 nmol/mL, *p* = 0.015). There was no effect of treatment, sex or treatment × sex interaction on the activity of other antioxidant indicators in the serum of pigs (*p* > 0.05).

**Table 2:** Effects of herbal mixture on antioxidant indicators and IgM concentrations in serum of weaned pigs^1^.

**Items**	**Dietary treatment** ^**2**^								**SEM^3^**	***p*** **-value**		
	**Male**				**Female**							
	**CON**	**HM1**	**HM2**	**HM3**	**CON**	**HM1**	**HM2**	**HM3**		**Treatment**	**Sex**	**Treatment × sex**
**Day 14**												
SOD (U/mL)	155.14^b^	166.68^a^	160.95^ab^	165.82^ab^	164.10^ab^	156.08^ab^	167.45^a^	161.34^ab^	4.10	0.656	0.974	0.060
CAT (U/mL)	5.62^ab^	5.22^b^	6.05^a^	5.84^ab^	5.39^ab^	6.05^ab^	5.75^ab^	6.10^a^	0.20	0.243	0.458	0.106
GSH (μmol/L)	58.47^b^	59.82^ab^	64.67^a^	64.92^a^	62.25^ab^	63.74^ab^	58.43^b^	64.20^ab^	2.02	0.239	0.899	0.052
GSH-PX (U/mL)	642.83^ab^	709.24^a^	681.08^a^	653.01^ab^	629.52^ab^	592.77^b^	598.75^b^	661.54^ab^	29.06	0.867	0.016	0.117
T-AOC (U/mL)	5.63^bc^	5.65^bc^	5.66^abc^	5.74^a^	5.60^c^	5.67^abc^	5.66^abc^	5.71^ab^	0.029	0.011	0.780	0.861
MDA (nmol/mL)	3.46^a^	3.12^ab^	2.98^b^	3.01^b^	2.99^b^	3.06^b^	2.87^b^	2.97^b^	0.13	0.149	0.075	0.368
IgM (mg/mL)	1.86^b^	1.89^b^	1.93^a^	1.89^b^	1.86^b^	1.93^a^	1.90^ab^	1.92^a^	0.02	0.026	0.645	0.187
**Day 28**												
SOD (U/mL)	169.65	175.20	180.48	182.01	172.75	172.67	169.55	173.45	9.17	0.914	0.469	0.869
CAT (U/mL)	5.95^b^	7.07^a^	7.03^a^	7.01^a^	5.99^b^	6.46^ab^	6.76^ab^	6.58^ab^	0.31	0.022	0.172	0.765
GSH (μmol/L)	63.02	66.51	66.54	66.98	65.36	66.06	64.63	63.97	2.19	0.805	0.639	0.640
GSH-PX (mg/mL)	650.53	641.22	622.65	664.21	646.32	660.04	675.39	603.67	26.42	0.925	0.932	0.250
T-AOC (mmol/gprot)	4.35^b^	4.75^a^	4.54^ab^	4.57^ab^	4.38^b^	4.61^ab^	4.79^a^	4.60^ab^	0.11	0.018	0.770	0.401
MDA (nmol/mL)	3.54^a^	3.23^abc^	3.17^abc^	2.99^c^	3.45^ab^	3.29^abc^	3.06^bc^	2.93^c^	0.15	0.015	0.679	0.952
IgM (mg/mL)	1.92	1.93	1.94	1.94	1.93	1.94	1.93	1.92	0.010	0.740	0.753	0.290

*Least-square means ± S.E.M*.

*SOD, superoxide dismutase; CAT, catalase; GSH, glutathione; GSH-PX, glutathione peroxidase; T-AOC, total antioxidant capacity; MDA, malondialdehyde; IgM, immunoglobulin M*.

*^1^Data were the mean of 16 replicates per treatment*.

*^2^Dietary treatments: CON, the control group, fed with the basal diet; HM1, the herbal mixture group 1, fed with the basal diet supplemented with 0.5 g herbal mixture/kg diet; HM2, the herbal mixture group 2, fed with the basal diet supplemented with 1.0 g herbal mixture/kg diet; HM3, the herbal mixture group 3, fed with the basal diet supplemented with 1.5 g herbal mixture/kg diet*.

*^3^SEM = Standard error of the mean*.

*^a−c^ Values in the same row not sharing a common superscript mean a significant difference (p < 0.05)*.

### Complete blood count test

The effects of the herbal mixture on the complete blood count of weaned pigs were presented in Supplementary Table 2. On day 14, female pigs had a higher concentration of MCHC compared with male pigs (317.91 vs. 309.97 g/L, *p* = 0.008). On day 28 of the trial, dietary supplementation with herbal mixture had greater number of WBC compared with pigs offered the basal diets (23.13, 24.32, and 23.98 vs. 22.60 × 10^9^/L, *p* < 0.001). The number of NEU in the HM2 and HM3 groups were significantly greater than that in the CON group (12.36 and 11.98 vs. 10.17 × 10^9^/L, *p* = 0.001). Similarly, the number of MON in the HM2 and HM3 groups were significantly greater than that in the CON group (1.97 and 2.04 vs. 1.70 × 10^9^/L, *p* < 0.001). Dietary supplementation with herbal mixture had greater concentration of MCHC compared with pigs offered the basal diets (328.81, 319.69, and 323.81 vs. 313.31 g/L, *p* < 0.001). The number of PCT in the HM1 group was significantly less than that in the CON group (0.31 vs. 0.45%, *p* = 0.025).

### Effect of herbal mixture on intestinal microbiota

To evaluate the effects of the herbal mixture on intestinal microbiota diversity, 16s rRNA gene sequencing was performed using the fresh feces of weaned pigs. A total of 7,205,863 clean reads were obtained from 96 fecal samples after quality control of the original raw reads with an average of 75,061 ± 1,233 reads per sample (Supplementary Table 3). These results indicated that the sequencing data met the criteria for further analysis.

### Effect of herbal mixture on intestinal microbiota diversity of weaned pigs

**Figure 1:**
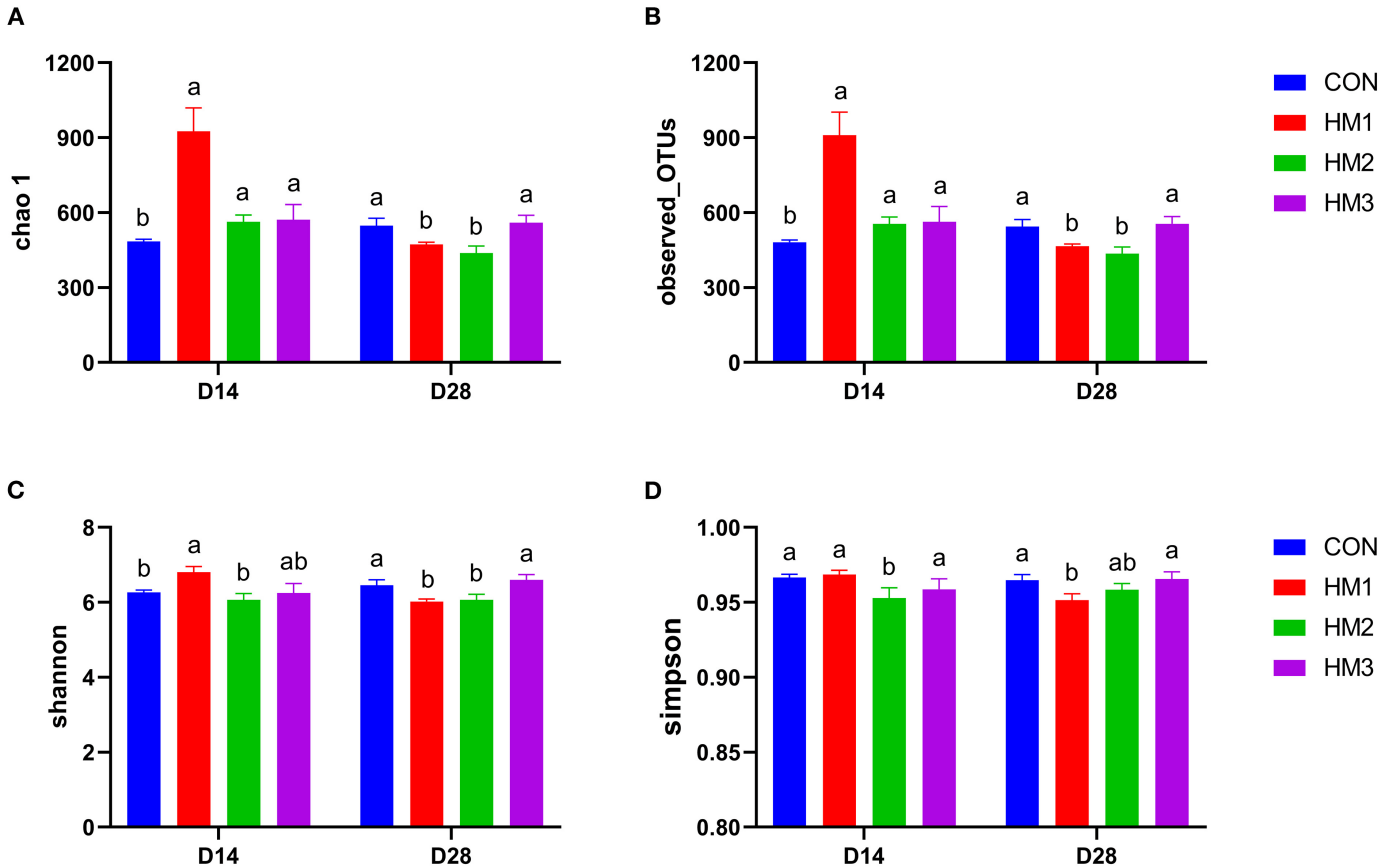
Effects of herbal mixture on the intestinal microbiota diversity in weaned pigs on day 14 and day 28 based on alpha diversity parameters of chao1 index **(A)**, observed OTUs **(B)**, shannon index **(C)** and simpson index **(D)**. The X-axis is the experimental period, and the Y-axis is the diversity indexes. CON, basic diet; HM1, basic diet with 0.5 g/kg of herbal mixture; HM2, basic diet with 1.0 g/kg of herbal mixture; HM3, basic diet with 1.5 g/kg of herbal mixture. Different lowercase letters in the figure indicate statistically significant differences (*p* < 0.05).

As shown in Figure 1, chao 1, observed_OTUs, shannon, and simpson indexes were used to analyze the differences in Alpha diversity between groups. On day 14, chao1 and observed_OTUs indexes of the HM1, HM2, and HM3 groups were greater than those of the CON group (*p* < 0.05). The Shannon index of the HM1 group was greater than that of the CON group (*p* < 0.05). The Simpson index in the HM2 group was less than that in the CON group (*p* < 0.05). On day 28, chao1, observed_ OTUs, and Shannon index of the HM1 and HM2 groups were less than those of the CON group (*p* < 0.05). The Simpson index of the HM1 group was less than that of the CON group (*p* < 0.05).

### Effect of herbal mixture on intestinal microbiota composition and abundance in weaned pigs

**Figure 2:**
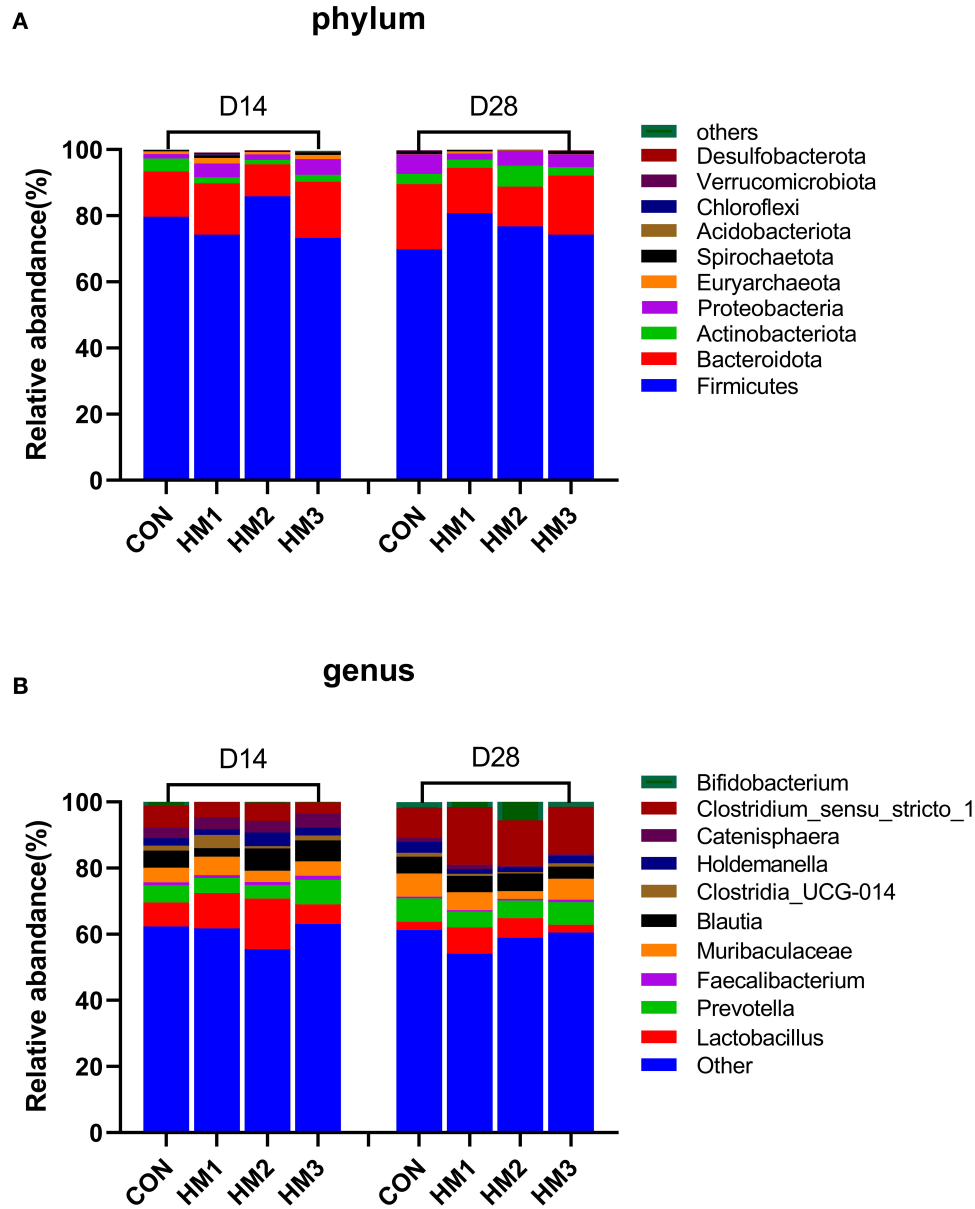
Intestinal microbiota compositions of different groups at phylum **(A)** and genus **(B)** levels on days 14 and 28. CON, basic diet; HM1, basic diet with 0.5 g/kg of herbal mixture; HM2, basic diet with 1.0 g/kg of herbal mixture; HM3, basic diet with 1.5 g/kg of herbal mixture.

The overall microbiota composition at the phylum and genus levels were presented in Figure 2. At the phylum level, the top 10 dominant bacterial species in the feces of weaned pigs were *Firmicutes, Bacteroidata, Actinobacteriota, Proteobacteria, Euryarchaeota, Spirochaetoa, Acidobacteriota, Chloroflexi, Verrucomicrobiota*, and *Desulfobacterota*. The fecal flora composition of the pigs was dominated by *Firmicutes*, accounting for 78.29%, and 75.44% on day 14, and 28, respectively. *Bacteroidata* accounted for 13.96%, and 15.79% on day 14 and 28, respectively (Figure 2A). At the genus level, the top 10 dominant bacterial species in the feces of weaned pigs were *Lactobacillus, Bifidobacterium, Clostridium_sensu_stricto_1, Prevotella, Muribaculaceae, Blautia, Clostridia_UCG-014, Catenisphaera, Holdemanella*, and *Faecalibacterium*. The fecal flora composition of the pigs was dominated by *Lactobacillus*, accounting for 9.79%, and 4.70% on day 14, and 28, respectively (Figure 2B).

**Figure 3:**
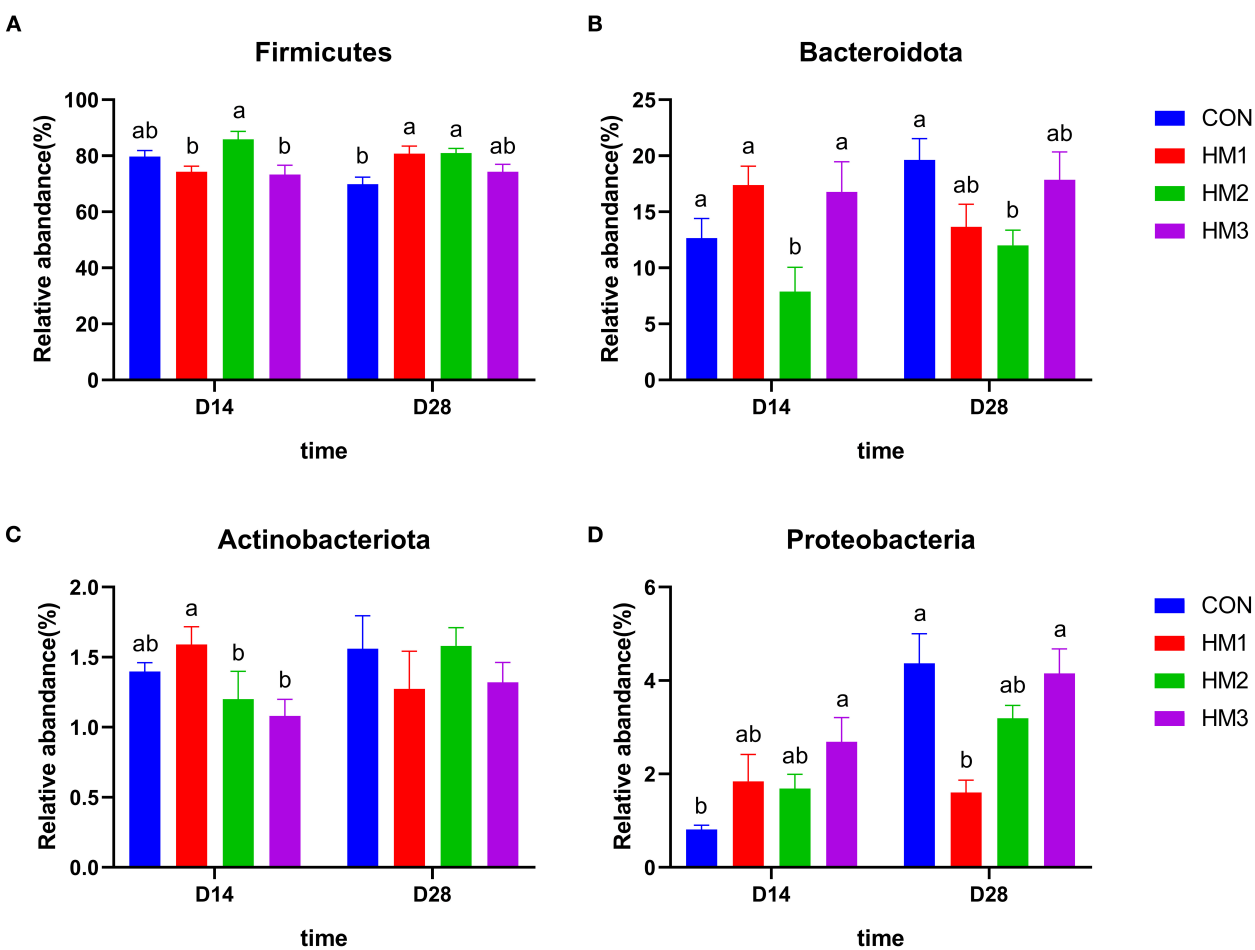
Comparison of dominant intestinal microbiota at phylum level. Relative abundances of *Firmicutes* **(A)**, *Bacteroidetes* **(B)**, *Actinobacteria* **(C)**, and *Proteobacteria* **(D)** among the four groups on d 18 and d 35. CON, basic diet; HM1, basic diet with 0.5 g/kg of herbal mixture; HM2, basic diet with 1.0 g/kg of herbal mixture; HM3, basic diet with 1.5 g/kg of herbal mixture. Different lowercase letters in the figure indicate statistically significant differences (*p* < 0.05).

When comparing the relative abundance of the four dominant phyla of the intestinal microbiota between groups (Figure 3), we found that there were significant differences between the experimental groups and the control group. On day 14, the relative abundance of *Firmicutes* in the HM2 group was greater than that in the HM1 and HM3 groups (*p* < 0.05) (Figure 3A). The relative abundance of *Bacteroidata* in the HM2 group was less than that in the CON group (*p* < 0.05) (Figure 3B). The relative abundance of *Actinobacteriota* in the HM1 group was greater than that in the HM2 and HM3 groups (*p* < 0.05) (Figure 3C). The relative abundance of *Proteobacteria* in the HM3 group was greater than that in the CON group (*p* < 0.05) (Figure 3D). Male pigs had a lower relative abundance of *Firmicutes* compared with female pigs (74.59 vs. 81.98%, *p* = 0.009), while male pigs had a higher relative abundance of *Bacteroidata* (16.80 vs. 10.80%, *p* = 0.019) and *Proteobacteria* (2.41 vs. 1.33%, *p* = 0.006) compared with female pigs (Supplementary Figure 1). There was a significant effect of treatment × sex interaction on the relative abundance of *Actinobacteriota* and *Proteobacteria* on day 14 (*p* < 0.05). Specifically, the relative abundance of *Actinobacteriota* in the HM2 group was increased compared with the HM3 group in the male pigs (1.80 vs. 1.13%, *p* = 0.002). In the female pigs, the relative abundance of *Actinobacteriota* in the HM2 group was decreased compared with the HM1 group (0.70 vs. 1.70%, *p* = 0.002) and CON group (0.70 vs. 1.45%, *p* = 0.002) (Supplementary Figure 2A). In the male pigs, the relative abundance of *Proteobacteria* in the HM1 and HM3 groups were greater than that in the CON group (3.70 vs. 0.76%, 3.79 vs. 0.76%, *p* = 0.001, respectively). In the female pigs, the relative abundance of *Proteobacteria* in the HM2 group was greater than that in the CON group (1.98 vs. 0.83%, *p* = 0.001) (Supplementary Figure 2B). On day 28, the relative abundance of *Firmicutes* in the HM1 and HM2 groups was greater than that in the CON group (*p* < 0.05) (Figure 3A). The relative abundance of *Bacteroidata* in the HM2 group was less than that in the CON group (*p* < 0.05) (Figure 3B). The relative abundance of *Proteobacteria* in the HM1 group was less than that in the CON group (*p* < 0.05) (Figure 3D). Male pigs had a higher relative abundance of *Firmicutes* (80.79 vs. 72.35%, *p* = 0.009), while had a lower relative abundance of *Bacteroidata* (12.36 vs. 19.22%, *p* = 0.009) compared with female pigs (Supplementary Figure 1). In the male pigs, the relative abundance of *Proteobacteria* in the HM1 group was less than that in the CON group (1.35 vs. 4.63%, *p* = 0.025) (Supplementary Figure 2B).

**Figure 4:**
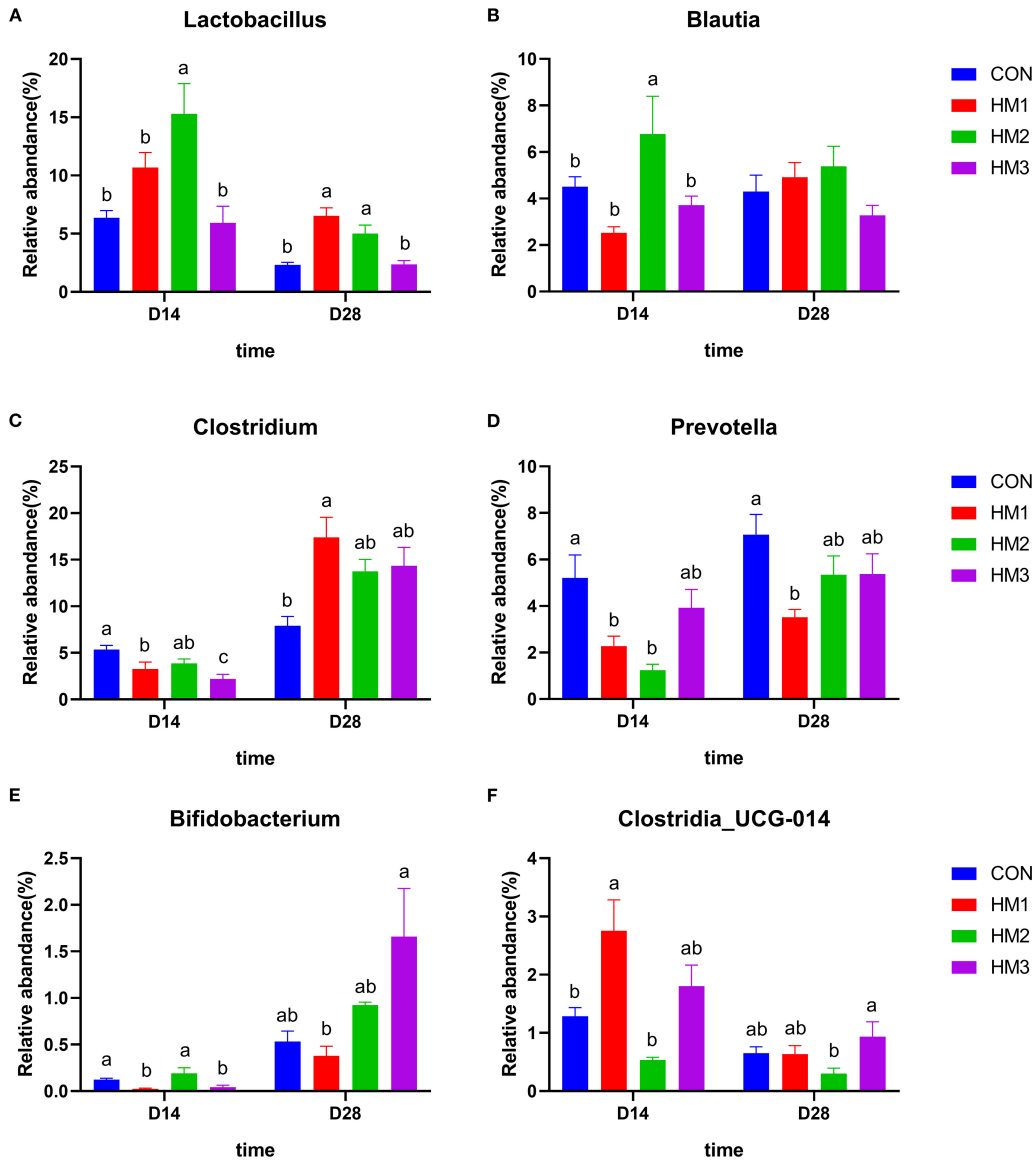
Comparison of dominant intestinal microbiota at genus level. Relative abundances of *Lactobacillus* **(A)**, *Clostridium* **(B)**, *Blautia* **(C)**, *Clostridia_UCG-014* **(D)**, *Prevotella* **(E)**, and *Bifidobacterium* **(F)** among the four groups on d 18 and d 35. CON, basic diet; HM1, basic diet with 0.5 g/kg of herbal mixture; HM2, basic diet with 1.0 g/kg of herbal mixture; HM3, basic diet with 1.5 g/kg of herbal mixture. Different lowercase letters in the figure indicate statistically significant differences (*p* < 0.05).

The relative abundance of the dominant genus of the intestinal microbiota between groups were presented in Figure 4. On day 14, the relative abundance of *Lactobacillus* and *Blautia* in the HM2 group was greater than that in the other groups (*p* < 0.05) (Figures 4A,B). The relative abundance of *Clostridium* and *Prevotella* in the HM groups was less than that in the CON groups (*p* < 0.05) (Figures 4C,D); The relative abundance of *Bifidobacterium* in the HM1 and HM3 groups were less than that in the CON group (*p* < 0.05) (Figure 4E); The relative abundance of *Clostridia_UCG-014* in the HM1 group was greater than that in the CON group (*p* < 0.05) (Figure 4F). In the male pigs, the relative abundance of *Bifidobacterium* in the HM2 group was greater than that in the CON group (0.29 vs. 0.11%, *p* = 0.008) (Supplementary Figure 3). On day 28, the relative abundance of *Lactobacillus* in the HM1 and HM2 groups was greater than that in the CON group (*p* < 0.05) (Figure 4A). The relative abundance of *Clostridium* in the HM1 group was greater than that in the CON group (*p* < 0.05) (Figure 4C). The relative abundance of *Prevotella* in the HM1 group was less than that in the CON group (*p* < 0.05) (Figure 4D). Female pigs had a higher relative abundance of *Prevotella* (6.36 vs. 4.38%, *p* = 0.014) compared with female pigs (Supplementary Figure 4).

**Figure 5:**
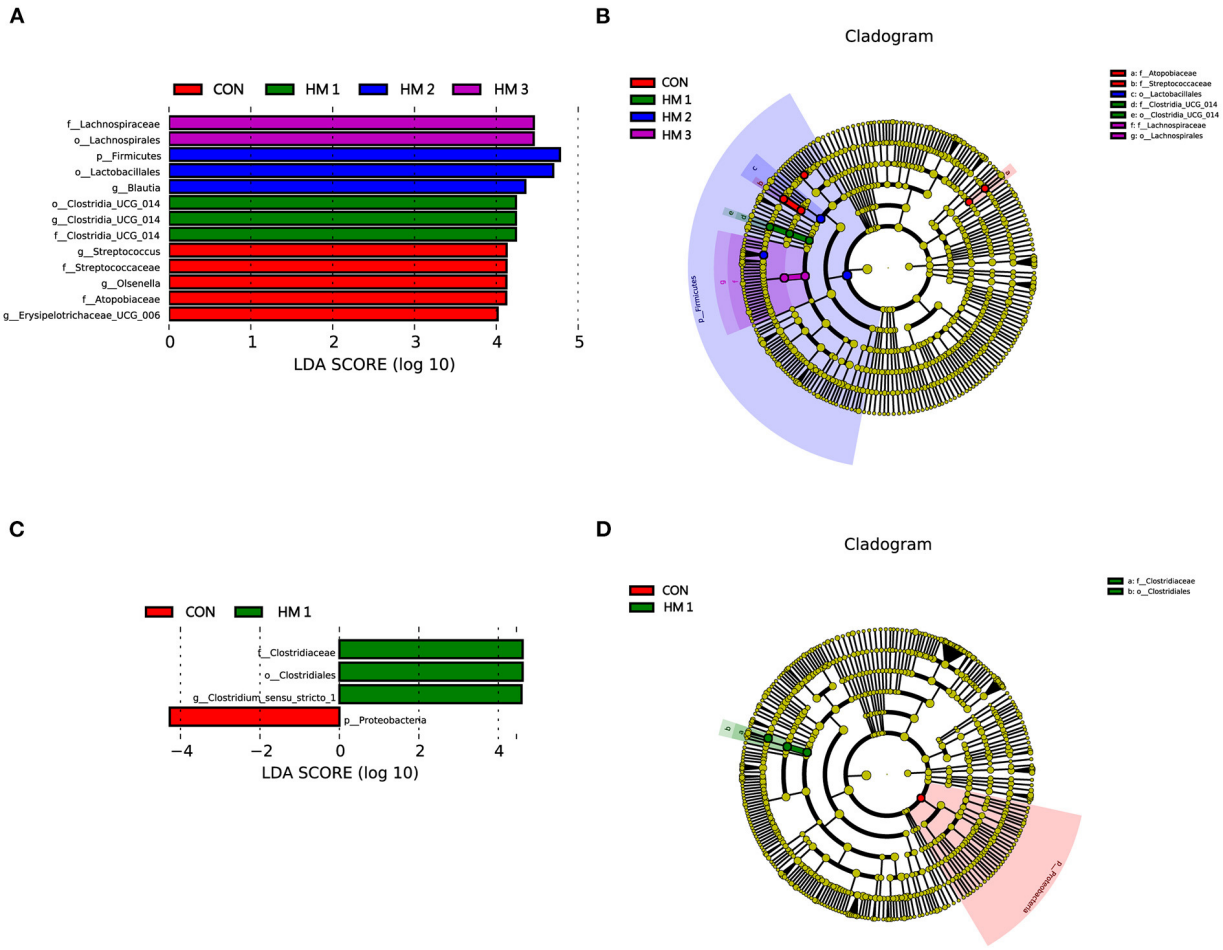
Linear discriminant analysis coupled with effect size (LEFse). The LEfSe analysis identified the differentially abundant (LDA score > 4) bacterial taxa between the HM group and the CON group on day 14 **(A)** or on day 28 **(C)**. A linear discriminant analysis (LDA) score higher than 4 indicates that the relative abundance of the corresponding group is higher than that of the other groups. The abscissa of the bar chart represents the LDA value, and the ordinate is the different species of the selected treatments. Cladogram showing the most discriminative bacterial clades identified by LEfSe between the HM group and CON group on day 14 **(B)** or on day 28 **(D)**. LEfSe taxonomic clade: Different colors indicate that some taxa are enriched in different groups. The size of a circle is based on relative abundance. CON, basic diet; HM1, basic diet with 0.5 g/kg of herbal mixture; HM2, basic diet with 1.0 g/kg of herbal mixture; HM3, basic diet with 1.5 g/kg of herbal mixture.

To identify bacterial taxa that significantly differentiated among HM and CON groups, a linear discriminant analysis effect size (LEfSe) analysis (LDA score > 4) was performed (Figure 5). On day 14, *Clostridia_UCG-014* (order), *Clostridia_UCG-014* (genus), and *Clostridia_UCG-014* (family) were more abundant in the HM1 group; *Firmicutes* (phylum), *Lactobacillale*s (order), and *Blautia* (genus) were more abundant in the HM2 group; *Lachnospiraceae* (family), and *Lachnospirales* (order) were more abundant in HM3 group; *Streptococcus* (genus), *Streptococcaceae* (family), *Olsenella* (genus), *Atopobiaceae* (family), and *erysipelotrichaceae_UCG-006* (genus) were enriched in the CON group (Figure 5A). The cladogram reports the taxa showing different abundance values according to LEfSe. On day 14, 5 and 2 different taxonomic levels of microorganisms were identified as potential biomarkers between the HM and CON groups, respectively (Figure 5B). On day 28, the relative abundances of *Clostridiaceae* (family), *Clostridiales* (order), and *Clostridium*_*sensu*_*stricto*_*1* (family) increased significantly in the HM1 group, whereas *Proteobacteria* (phylum) was more prevalent in the CON group (Figure 5C). However, there were no significant differences in microbiota abundance between the HM2, HM3, and CON groups. On day 28, only 2 different taxonomic levels of microorganisms were identified as potential biomarkers in the HM1 group (Figure 5D).

### Effect of herbal mixture on intestinal microbiota function of weaned pigs

The Kyoto Encyclopedia of Genes and Genomes (KEGG) pathway enrichment analysis were presented in Supplementary Figure 5. The top 35 KEGG pathways between the HM and the CON groups were selected from the results of species function annotation and abundance information, and then clustered by their function annotation information and the inter-group differences, to obtain a KEGG pathway enrichment analysis heatmap. Predicted KEGG pathways were mainly concentrated in ABC-2 type transport system ATP-Binding protein, ATP-binding cassette, subfamily B, bacterial, sucrose-6-phosphatase, putative ABC transport system permease protein, ABC-2 type transport system permease protein, RNA polymerase sigma-70 factor, ECF subfamily, and K02003 putative ABC transport system ATP-binding protein pathways.

### Correlation analysis between the differential microbial species and measured parameters

**Figure 6:**
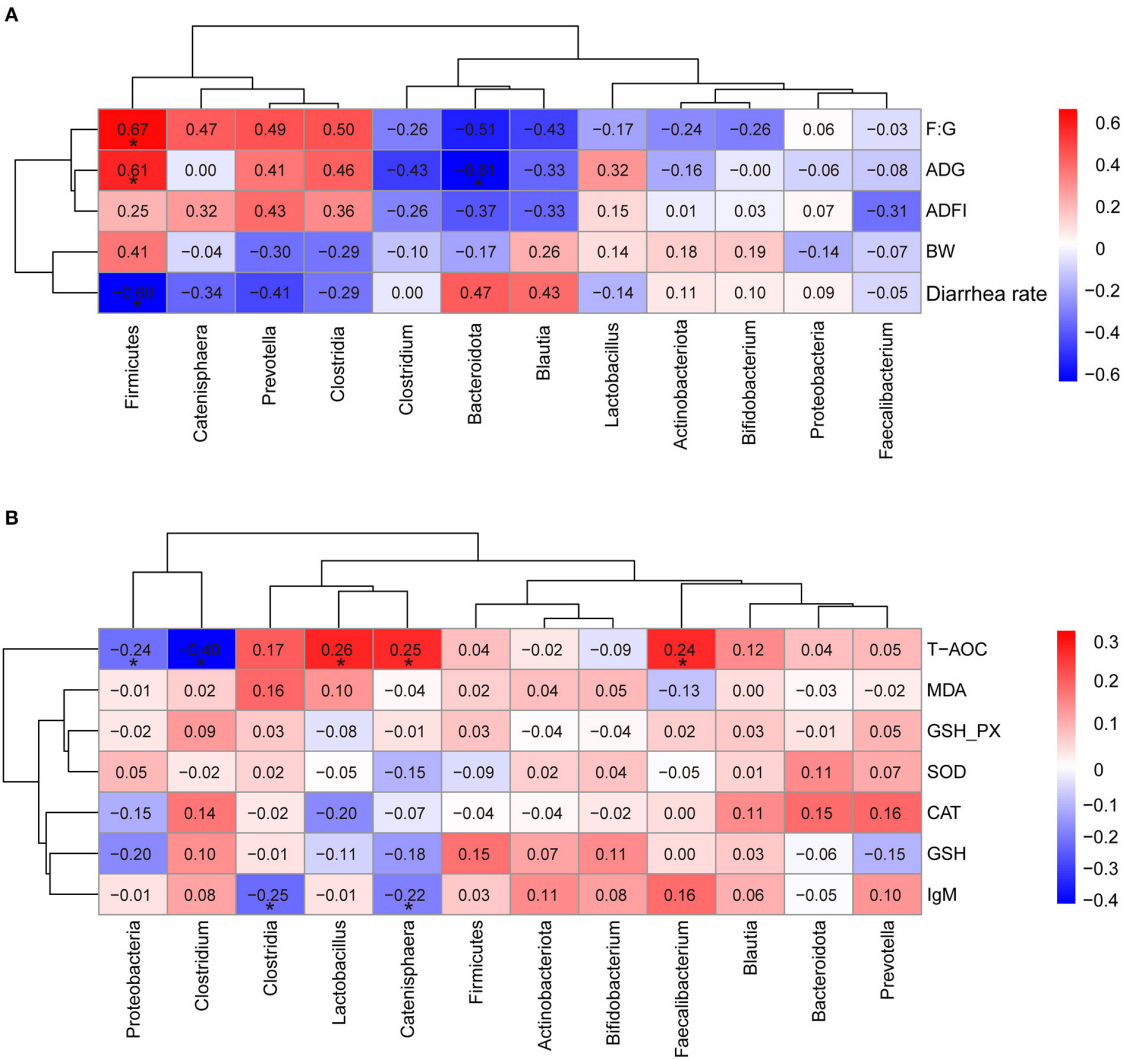
Heatmap of the partial correlation analysis between the dominant microbial species and growth performance **(A)**, serum antioxidant parameters **(B)**. ADG, the average daily gain; ADFI, average daily feed intake; F: G, feed to gain ratio; T-AOC, total antioxidant capacity; MDA, malondialdehyde; GSH-PX, glutathione peroxidase; SOD, superoxide dismutase; CAT, catalase; GSH, glutathione; IgM, immunoglobulin M. The red represents positive correlation and the blue represents negative correlation, respectively (*p < 0.05).

The partial correlation analyses between the relative abundance of dominant bacteria and growth performance, serum antioxidant parameters were presented in Figure 6. These results indicated that ADG and F: G were positively associated with phyla *Firmicutes* (R = 0.610, R = 0.674, respectively, *p* < 0.05) (Figure 6A). Whereas, ADG was negatively associated with phyla *Bacteroidota* (R = −0.609, *p* < 0.05) (Figure 6A). Meanwhile, the abundance of *Firmicutes* was negatively associated with the diarrhea rate of pigs (R = −0.600, *p* < 0.05). Interestingly, the serum T-AOC were positively associated with genus *Lactobacillus, Catenisphaera*, and *Faecalibacterium* (R = 0.256, R = 0.247, R = 0.244, respectively, *p* < 0.05), while negatively associated with phyla *Proteobacteria* and genus *Clostridium* (R = −0.239, R = −0.399, respectively, *p* < 0.05) (Figure 6B). Furthermore, *Clostridia* and *Catenisphaera* had significant negative correlation with the concentration of serum IgM (R = −0.251, R = −0.222, respectively, *p* < 0.05) (Figure 6B).

## Discussion

Herbal medicine and their extracts are widely used as health promoters and novel approaches to disease treatment (35). Meanwhile, herbal medicine feed additives have also been receiving increasing attention due to their ability to improve the nutrition and wellbeing of farm animals (36). Furthermore, herbal medicine as an alternative to antibiotic feed additives improved the growth performance, meat quality, and nutrient digestibility parameters of pigs, which contributed to reducing the cost of raising pigs and produced antibiotic-free pork products (37). Combined herbal mixture have been shown to be more effective in improving animal health than single herbal extracts (38). Herbal mixtures are also regarded as one of the most economical and effective additives due to their ease of preparation and low cost, so their use has increased in livestock production (39). However, the effect of the herbal mixture containing Paeoniae Radix Alba, licorice, dandelion, and tea polyphenols on weaned pig production is not yet known. The present study aims to investigate the effects of these herbal mixtures on the growth performance, antioxidant capacity, immune response, and intestinal microbiota of weaned pigs.

In the swine industry, newly weaned pigs simultaneously encounter psychological, physiological, and nutritional pressures, resulting in impaired the digestive and absorptive capacity, which has a negative impact on feed intake and daily gain of weaned pigs (40). It is reported that adding feed additives to the diet reduces the negative effects of weaning stress (41). We first investigated the effect of herbal mixtures as promising feed additives that are able to substitute antibiotics on the growth performance of weaned pigs. Our findings indicated that the dietary supplementation of herbal mixture significantly improved the growth performance of weaned pigs as demonstrated by several indicators, including the BW, ADG, ADFI, and F: G, which suggested that herbal mixture effectively helped pigs overcome weaning stress. Specifically, the body weight at the end of the experiment period and the average daily gain during the whole experiment period of the pigs supplemented with herbal mixture were significantly greater than those of the CON group. Among them, the HM2 group (basic diet with 1.0 g/kg of herbal mixture) had the highest BW and ADG at the end of the trial. Previous studies found that weaned pigs fed herbal mixtures (including Codonopsis pilosula, Radix astragalus, R. isatidis, R. paeoniae alba, and Atractylodes macrocephalae) supplementation diets had positive effects on BW and ADG, which was in agreement with our results (42). We speculated that the beneficial effects of herbal mixtures on the growth performance of pigs due to a variety of nutrients and active ingredients in herbal medicines, which improved digestive tract function in pigs (43). In addition, we also observed that the feed intake of pigs in the HM1 and HM2 group were greater than that of CON group during the second experiment period, while the feed intake of pigs in the HM3 group was the lowest among the four groups during the whole experiment period. Due to herbal mixture ingredients including Paeoniae Radix Alba, dandelion, and tea polyphenols tasting bitter to mammals (44, 45), we speculated that the bitter taste of a high dose herbal mixture added to the diet might affect the appetite of pigs, resulting in a decrease in feed intake. Feed conversion efficiency is a very important indicator in pig production that determines the economic benefits of breeding. In the present study, the F: G of pigs supplemented with herbal mixture was significantly less than those in the CON group, which suggested that adding the herbal mixture to the diet was helpful to improve economic benefits. A previous study indicated that supplementation of plant extract mixtures in the drinking water increased ADG and decreased F: G in weaned pigs (46). Supplementation of 1.0 g dandelion/kg improved ADG, feed efficiency, and nutrient digestibility of weaned pigs (47). These were basically consistent with the experimental results presented in our study. The present study demonstrated that adding herbal mixture to the diets of pigs improved the growth performance of pigs, including improving average daily gain and reducing feed to weight ratio. Among them, dietary supplemented with 1.0 g/kg herbal mixture had the best effect.

Early weaned pigs are vulnerable to diarrhea and even death due to weaning stress and immature intestinal development (48). In the present study, the diarrhea rate of pigs supplemented with herbal mixture was significantly lower than that of the CON group, and the frequency of diarrhea decreased with the increase of the herbal mixture supplementation. A previous study reported that plant derived polyphenol extracts reduced post-weaning diarrhea caused by E. coli in pigs (49). A traditional herbal medicine called “Tongxie Yaofang” containing paeoniae lactiflora has been widely used to treat diarrhea-predominant irritable bowel syndrome (50). A herbal medicine compound containing licorice extract reduced diarrhea rates and improved the nutrient apparent digestibility of piglets (51). These observations were consistent with our results in the present study. The herbal mixture contains both Paeoniae Radix Alba, licorice and polyphenol extracts in this study, and their combination may be the reason for the significant reduction in diarrhea rate in weaned pigs.

Weaning stress disrupts free-radical metabolism and the antioxidative system, causing severe oxidative stress and mitochondrial dysfunction in pigs (52, 53). The serum antioxidant indicators like GSH, T-AOC, SOD, GSH-Px, and CAT were commonly used to assess pig antioxidant stress (54). The content of MDA reflects the degree of lipid peroxidation and impaired antioxidative capacity in animals (55). Next, we investigated the effects of the dietary supplementation of herbal mixture on antioxidant capacity of weaned pigs. In the present study, the concentrations of serum T-AOC and CAT in the herbal mixture groups were greater than that those in the CON group, whereas the concentration of serum MDA in herbal mixture group was lower. Single herbal medicine or herbal mixture extracts with antioxidant capacity have been widely used in both pharmaceutical raw materials and animal husbandry (56, 57). For example, a previous study demonstrated that an optimized mixture of plant polyphenols (apples, grape seeds, green tea, and olive leaves) increased serum GSH-Px and T-AOC activity of piglets (58). Similarly, supplementation of dandelion leaf powder increased the concentration of serum GSH-Px but decreased the concentration of MDA in broilers (59). Licorice extract, as the other ingredient in the mixture of herbal medicine, increased serum T-AOC level, and 50 mg/kg increased serum T-SOD activity and decreased serum MDA concentration in weaned pigs (60). It had been reported that paeoniflorin inhibits cell apoptosis, improves cell viability, and increases the concentration of SOD and CAT (61). These results demonstrated that the supplementation of herbal mixture to the diet decreased the oxidative stress level of weaned pigs, improved the antioxidant capacity of weaned pigs. This is probably due to the fact that the five herbs used in the herbal mixture in our study contain a variety of active ingredients with antioxidant effects.

Weaning stress causes immune system dysfunction and results in a negative impact on performance and health in pigs (62). IgM is the major serum immunoglobulin (63). The level of serum IgM was associated with the level of the autoimmune ability of piglets (64). White blood cells participate in the immune response of animals, and their abnormal increase indicates the presence of inflammation, so they are important clinical indicators for health status (65, 66). Both neutrophils and monocytes are immune-related white blood cells that aid in the host's defense against invading pathogens (67). We next further investigated the effect of adding herbal medicine mixture to the diet on the immune index of weaned pigs. In the present study, the serum IgM concentrations of pigs fed with the herbal mixture increased, indicating that the antibody produced by B lymphocytes was increased, which was conducive to improving the humoral immunity of pigs. Similarly, previous research has shown that supplementation with herbal mixture (include dandelion and licorice) increased serum IgG and IgM concentrations in piglets (68). This is basically consistent with our research results. It had been reported previously that herbs, such as Radix Alba (69), licorice (70), dandelion (71), and tea polyphenols (72), have functions of immunomodulatory and resisting inflammation, which could be used as a new-type immunoenhancer. The synergistic effect of Radix Alba and licorice improved immunity and reduced inflammation (73). Furthermore, our results indicated that the treatment of the herbal mixture with 1.0 and 1.5 g/kg concentrations significantly increased the WBC, NEU, and MON counts compared with the CON group. As immune biomarkers, abnormal levels of white blood cell (WBC) counts indicate abnormal immune processes (74). Neutrophils are the most abundant white blood cells and an important part of the innate immune system (75). Monocytes are innate immune cells and play an important defensive role in the primary innate immune response (76). A previous study had demonstrated that green tea extract increased the WBC counts in finishing pigs (77). WBC concentration was greater in pigs fed an herbal mixture (including: Astragalus membranaceus, Codonopsis pilosula and allicin) than in the CON group (16). This indicated that the herbal mixture had the potential to attenuate inflammation and enhance immunity in weaned pigs. Mean corpuscular hemoglobin concentration (MCHC) is a parameter of risk for anemia in weaning pigs (78). In the present study, the mean corpuscular hemoglobin concentration in the HM groups was greater than that of in the CON group, indicating that herbal mixture could reduce the incidence of anemia in weaned pigs by increasing corpuscular hemoglobin concentration. Based on the improvement of immunity indicators that accompanied supplementation with herbal mixture, we speculated that adding the herbal mixture to the feed could improve the swine immunity and also their general health status.

The gut microbiota is essential for gut homeostasis and host health (79). When people investigated the microbiota associated with health and diseases, they found that microbial diversity ensures the consistent immune regulation ability of microbiota, so it is more important to understand microbial diversity (80). Microbial diversity was assessed using the Chao1, observed_OTUs, Shannon, and Simpson index (81). In the present study, chao 1 and observed_OTUs in the HM groups were greater than those in the CON group on day 14 of the experiment. In contrast, chao 1, observed_OTUs, and Shannon index in the HM groups were less than those in the CON group on day 28 of the trial period. This indicates that the herbal mixture significantly improved the diversity of bacterial community in the early stage of the experiment. As animals aged, the number of microbial species in the feces of weaned pigs decreased after adding an herbal mixture to the diet. A previous study reported that the alpha diversity indices of intestinal microbiota in pigs changed with age, and they decreased at 35 days after weaning compared with 21 days after weaning (42). We speculated that the addition of herbal mixtures to the diet might inhibit pathogenic bacteria colonization in the intestine over time, and then reduce the diversity of intestinal flora.

In addition to its role in microbial diversity, our study also indicated that the addition of herbal mixtures to the diet had an impact on the abundance of intestinal flora in piglets. In this experiment, *Firmicutes* and *Bacteroidetes* were the dominant phyla in the gut microbiome of piglets, which was basically consistent with previous report (82). We also found that the HM2 group (basic diet with 1.0 g/kg of herbal mixture) significantly increased the relative abundance of *Firmicutes*, while decreasing the relative abundance of *Bacteroidetes* compared to the CON group. Meanwhile, the HM2 group had the highest BW and ADG at the end of the trial. This indicated obese pigs had a higher abundance of *Firmicutes* and a lower abundance of *Bacteroidetes* than lean pigs during the nursery period. Human and mouse studies yielded similar results (83, 84). In addition, the abundance of *Firmicutes* and *Bacteroidetes* was related to fat deposition in animals (85, 86). These results suggested that the intestinal microbiota (i.e., *Firmicutes, Bacteroides*) affected by the herbal mixture might affect the fat metabolism of the host, leading to fat deposition and body weight gain. Furthermore, the pathogenic bacteria decreased in the herbal mixture groups. For example, the relative abundance of *Proteobacteria* was lower in the HM2 group than in the control group on day 28. Studies had revealed that plant-derived polyphenols reduce the relative abundance of *Proteobacteria* in pigs, attenuating diarrhea and intestinal damage (87). Our study also found that the diarrhea rate of HM2 group was significantly lower than that of the control group. These results suggested that changes in the abundance of *Proteobacteria* in the intestine might disrupt the balance of microbial community structure and the health state of the host. Supplementing the diet with herbal mixtures was able to reduce the risk of intestinal diseases. *Lactobacillus*, as a beneficial bacteria, maintains the balance of intestinal microflora and enhances the human immune function (88). In the present study, the relative abundance of *Lactobacillus* in the HM1 and HM2 groups were greater than that in the CON group. Therefore, there was reason to believe that supplementing an appropriate dose of herbal mixture improved the relative abundance of *Lactobacillus*, which was conducive to improving host health. Interestingly, we found that *Lactobacillus* decreased but *Bifidobacterium* increased in the group with 1.5 g/kg herbal mixture. Previous studies showed that *Lactobacillus* and *Bifidobacterium* strains had different effects on host inflammation and intestinal microbial fermentation and composition (89). Lactic acid inhibits the proliferation of *Lactobacillus* during the fermentation process (90). Therefore, we speculate that the addition of high-dose herbal mixtures to the feed produce more lactic acid in the intestine, which inhibits the abundance of *Lactobacillus* in the intestine. Since *Lactobacillus* and *Bifidobacterium* are two major probiotics in the intestine (91), the abundance of *Bifidobacterium* could increase to maintain the balance of probiotics when the abundance of *Lactobacillus* decreases. The function prediction of the microbiota analysis suggested that the main pathways were protein transport, ATP binding, and sucrase, which indirectly reflected the microorganisms participate in the digestion and absorption of nutrients in the pig intestine. Our findings indicated that supplementing with herbal mixture to the diet had an inhibitory effect on pathogenic bacteria like *Proteobacteria* while increasing the relative abundance of beneficial bacteria like *Lactobacillus* and ultimately helping pigs cope with weaning stress.

When there are multiple variables, the correlation analysis between the two variables is often affected by other variables (92). In order to exclude the possible influence of controlling variables on other related variables, partial correlation analysis was used to assess the correlation between the relative abundance of dominant bacteria and measured parameters. Partial correlation analysis indicated that ADG was significantly positively correlated with the abundance of *Firmicutes* and negatively correlated with the abundance of *Bacteroides*. Previous reports indicated that the ADG of meat goats was linearly associated with the abundance of *Firmicutes* (93), which was consistent with our experimental observations. In this study, we did not find a significant correlation between body weight and the abundance of *Firmicutes*, but there was a positive correlation (R = 0.41). We speculated that the selection of samples and the differences between individuals mask this correlation due to limited sample sizes. The abundance of *Firmicutes* and *Bacteroides* has the potential to be considered as a biomarker to evaluate the body weight gain performance of pigs. Due to pigs being closer to humans in genetics, anatomy, and physiology and often used as biomedical models (94), our findings also support the view of *Firmicutes* and *Bacteroidetes* to evaluate human obesity (95). Our study also found that the abundance of *Lactobacillus* was positively associated with the T-AOC activity in the serum. *Lactobacillus* has been shown to have an antioxidant effect and to reduce oxidative stress injury by lowering ROS level (96). This indicated that supplementing herbal mixtures to the diet was capable of enhancing the abundance of *Lactobacillus* and then improving the antioxidant capacity of pigs.

## Conclusions

The current findings indicated that supplementation of the herbal mixture (including: Paeoniae Radix Alba, licorice, dandelion, and tea polyphenols) to the diet significantly improved the ADG, while reduced feed to gain ratio (F: G) of weaned pigs. Our results also demonstrated that the herbal mixture significantly improved the total antioxidant capacity of weaned pigs. In addition, we also found that the relative abundance of beneficial intestinal microbiota such as *Lactobacillus* was increased in the pigs supplemented with 1.0 g/kg herbal mixture to the diet. These results also demonstrated the potential effect of the herbal mixture as an alternative to antibiotics in promoting the growth of weaned pigs, improving the ability of antioxidant capacity, and maintaining the intestinal microflora balance and improving the overall health of the host.

## Data availability statement

The data presented in the study are deposited in the NCBI repository, accession number PRJNA851026. We have release our data, the data can be found at: https://www.ncbi.nlm.nih.gov/bioproject/PRJNA851026.

## Ethics statement

The animal study was reviewed and approved by the Animal Care and Use Committee of Nanjing Agricultural University (SYXK2017-0027, Nanjing, China). Written informed consent was obtained from the owners for the participation of their animals in this study.

## Author contributions

QX and MC analyzed the data. QX wrote—review and editing and visualization. MC wrote—original draft and investigation. RJ provided the resources. MC, XZ, and ML conducted the animal experiments. JZ contributed to formal analysis. XC and CZ contributed to software and visualization. BZ contributed to the project administration, and funding acquisition, project supervision. All authors have read and approved the final version of the manuscript.

## Funding

This work was supported by the JBGS Project in Jiangsu Province, China (No. JBGS [2021]101).

## Conflict of interest

Author RJ was employed by Wuxi Sanzhi Bio-Tech Co., Ltd. The remaining authors declare that the research was conducted in the absence of any commercial or financial relationships that could be construed as a potential conflict of interest.

## Publisher's note

All claims expressed in this article are solely those of the authors and do not necessarily represent those of their affiliated organizations, or those of the publisher, the editors and the reviewers. Any product that may be evaluated in this article, or claim that may be made by its manufacturer, is not guaranteed or endorsed by the publisher.

## Abbreviations

ADG: average daily gain
ADFI: average daily feed intake
F: G: feed to gain ratio
BW: body weight
CAT: catalase
T-AOC: total antioxidant capacity
MDA: malondialdehyde
SOD: superoxide dismutase
GSH: glutathione
GSH-Px: glutathione peroxidase
HM: herbal mixture
CBC: complete blood count
IgM: immunoglobulin M
OTUs: Operational Taxonomic Units
SEM: standard error of the mean
CON: control
KEGG: Kyoto Encyclopedia of Genes and Genomes.
