# Supplementary material for: Effects of dietary supplement with a Chinese herbal mixture on growth performance, antioxidant capacity, and gut microbiota in weaned pigs
Source: Front Vet Sci. 2022 Aug 22;9:971647. doi: 10.3389/fvets.2022.971647 (PMC9442064; doi:10.3389/fvets.2022.971647)
Supplement: Supplementary file 1 [file Table_1.DOCX]

Supplementary Material

# Supplementary Table 1. Composition and nutrient levels of the basal diet (as fed basis).

| **Items** | **Period (8 - 18 kg)** | **Period (18 - 35 kg)** |
| --- | --- | --- |
| Ingredients (%) |  |  |
| Maize | 67.00 | 63.20 |
| Soybean meal | 18.00 | 20.50 |
| Wheat bran | 7.40 | 7.80 |
| Fish meal | 3.00 | 3.10 |
| Dicalcium phosphate | 1.40 | 1.80 |
| Limestone | 0.90 | 1.20 |
| Salt | 0.30 | 0.30 |
| Premix^1^ | 2.00 | 2.10 |
| Total | 100.00 | 100.00 |
| Nutrient levels (%) |  |  |
| Metabolisable energy (MJ/kg) | 14.10 | 13.50 |
| Crude protein | 20.00 | 19.00 |
| Lysine | 1.45 | 1.35 |
| Crude fiber | 4.00 | 5.00 |
| Crude ash | 7.00 | 7.00 |
| Phosphorus | 0.55 | 0.50 |
| Calcium | 0.70 | 0.60 |
| NaCl | 0.30 | 0.30 |

^1^ Premix were fed the following diets per kg: vitamin A 20 000 IU; vitamin D 2 000 IU; vitamin E 60 IU; vitamin K 2 mg; vitamin B_1_ 2 mg; vitamin B_2_ 10 mg; vitamin B_3_ 50 mg; vitamin B_5_ 20 mg; vitamin B_6_ 5 mg; vitamin B_12_ 40 μg; vitamin B_9_ 1.5 mg; vitamin B_7_ 0.15 mg; vitamin C 200 mg; vitamin B_4_ 600 mg; Mn 75 mg; Zn 120 mg; Fe 140 mg; Cu 8 mg; I 0.4 mg; Se 0.3 mg.
