# Supplementary material for: Effects of dietary supplement with a Chinese herbal mixture on growth performance, antioxidant capacity, and gut microbiota in weaned pigs
Source: Front Vet Sci. 2022 Aug 22;9:971647. doi: 10.3389/fvets.2022.971647 (PMC9442064; doi:10.3389/fvets.2022.971647)
Supplement: Supplementary file 2 [file Table_2.DOCX]

Supplementary Material

# Supplementary Table 2. Effects of herbal mixture on complete blood count.^1^ (Least-square means ± S.E.M.).

| **Items** | **Dietary treatment^2^** | | | | | | | | | **SEM^3^** | ***p*-value** | | | **Reference ranges** |
| --- | --- | --- | --- | --- | --- | --- | --- | --- | --- | --- | --- | --- | --- | --- |
|  | **Male** | | | | | **Female** | | | |  |  | | |  |
|  | **CON** | **HM1** | **HM2** | **HM3** | **CON** | | **HM1** | **HM2** | **HM3** |  | **Treatment** | **Sex** | **Treatment**  **× Sex** |  |
| Day 14 | | | | | | | | | | | | | | |
| WBC (10^9^/L) | 18.69 | 21.70 | 18.91 | 26.37 | 22.79 | | 22.51 | 24.18 | 18.93 | 4.03 | 0.970 | 0.812 | 0.414 | 10.20-30.00 |
| NEU (10^9^/L) | 8.36 | 9.57 | 7.22 | 9.91 | 8.15 | | 7.14 | 6.87 | 7.30 | 1.03 | 0.483 | 0.065 | 0.509 | 2.80-16.10 |
| LYM (10^9^/L) | 8.40^ab^ | 9.87^ab^ | 10.36^ab^ | 11.62^a^ | 10.81^ab^ | | 9.93^ab^ | 9.78^ab^ | 8.18^b^ | 1.14 | 0.982 | 0.634 | 0.096 | 4.80-16.20 |
| MON (10^9^/L) | 1.60^ab^ | 1.86^ab^ | 1.49^b^ | 2.30^a^ | 1.90^ab^ | | 2.04^ab^ | 2.22^ab^ | 1.57^ab^ | 0.27 | 0.872 | 0.538 | 0.062 | 0.20-2.25 |
| EOS (10^9^/L) | 0.46 | 0.39 | 0.31 | 0.35 | 0.46 | | 0.37 | 0.46 | 0.44 | 0.063 | 0.595 | 0.246 | 0.577 | 0.00-1.80 |
| BAS (10^9^/L) | 0.11^ab^ | 0.09^b^ | 0.08^b^ | 0.08^b^ | 0.093^ab^ | | 0.14^a^ | 0.12^ab^ | 0.094^ab^ | 0.017 | 0.671 | 0.112 | 0.124 | 0.00-0.46 |
| RBC (10^12^/L) | 5.75 | 6.11 | 6.14 | 5.79 | 5.66^b^ | | 6.32^a^ | 5.67^b^ | 5.63^b^ | 0.22 | 0.077 | 0.404 | 0.485 | 5.50-9.00 |
| HGB (g/L) | 96.88^b^ | 101.38^ab^ | 101.50^ab^ | 100.38^ab^ | 97.25^b^ | | 108.50^a^ | 100.00^ab^ | 97.38^b^ | 3.31 | 0.116 | 0.750 | 0.444 | 100.00-160.00 |
| HCT % | 30.81^b^ | 33.10^ab^ | 33.13^ab^ | 32.04^ab^ | 31.06^b^ | | 34.20^a^ | 31.39^ab^ | 30.28^b^ | 1.09 | 0.064 | 0.490 | 0.463 | 33.0-52.0 |
| MCV (fL) | 53.64 | 54.19 | 54.14 | 55.30 | 55.08 | | 54.23 | 55.46 | 53.94 | 1.01 | 0.936 | 0.616 | 0.473 | 51.0-73.0 |
| MCH (pg) | 16.83 | 16.58 | 16.60 | 17.34 | 17.31 | | 17.21 | 17.68 | 17.31 | 0.39 | 0.739 | 0.054 | 0.567 | 14.0-22.0 |
| MCHC (g/L) | 313.75^abc^ | 306.12^c^ | 306.62^bc^ | 313.38^abc^ | 314.25^abc^ | | 317.63^ab^ | 318.88^a^ | 320.87^a^ | 4.04 | 0.589 | 0.008 | 0.455 | 300.00-360.00 |
| PLT (10^9^/L) | 371.55^a^ | 255.25^ab^ | 352.13^a^ | 244.73^ab^ | 304.21^ab^ | | 315.75^ab^ | 230.19^b^ | 304.92^ab^ | 40.70 | 0.493 | 0.581 | 0.092 | 200.00-1000.00 |
| MPV (fL) | 9.18 | 8.93 | 9.58 | 9.04 | 8.85 | | 9.18 | 9.21 | 8.58 | 0.30 | 0.282 | 0.296 | 0.636 | 7.20-13.10 |
| PDW | 15.41 | 15.38 | 14.96 | 15.90 | 15.55 | | 15.41 | 15.59 | 15.41 | 0.25 | 0.499 | 0.665 | 0.197 | 12.00-17.50 |
| PCT % | 0.31^a^ | 0.23^abc^ | 0.34^a^ | 0.18^c^ | 0.25^abc^ | | 0.29^ab^ | 0.20^bc^ | 0.19^bc^ | 0.040 | 0.065 | 0.264 | 0.072 | 0.12-0.93 |
| Day 28 | | | | | | | | | | | | | | |
| WBC (10^9^/L) | 22.65^d^ | 23.27^bc^ | 24.35^a^ | 24.32^a^ | 22.55^d^ | | 22.99^cd^ | 24.30^a^ | 23.63^b^ | 0.20 | < 0.001 | 0.059 | 0.346 | 10.20-30.00 |
| NEU (10^9^/L) | 10.35^cd^ | 10.69^bcd^ | 12.71^a^ | 12.30^ab^ | 9.99^d^ | | 10.57^cd^ | 12.02^abc^ | 11.66^abc^ | 0.58 | 0.001 | 0.279 | 0.956 | 2.80-16.10 |
| LYM (10^9^/L) | 12.92^a^ | 10.21^ab^ | 9.88^b^ | 11.60^ab^ | 11.59^ab^ | | 12.56^ab^ | 10.13^ab^ | 9.58^b^ | 1.07 | 0.184 | 0.804 | 0.189 | 4.80-16.20 |
| MON (10^9^/L) | 1.67^c^ | 1.74^c^ | 1.97^ab^ | 2.03^a^ | 1.72^c^ | | 1.80^bc^ | 1.96^ab^ | 2.05^a^ | 0.060 | < 0.001 | 0.461 | 0.930 | 0.20-2.25 |
| EOS (10^9^/L) | 0.41 | 0.48 | 0.46 | 0.45 | 0.40 | | 0.31 | 0.47 | 0.38 | 0.076 | 0.799 | 0.284 | 0.690 | 0.00-1.80 |
| BAS (10^9^/L) | 0.08 | 0.07 | 0.08 | 0.08 | 0.10 | | 0.11 | 0.09 | 0.09 | 0.012 | 0.870 | 0.057 | 0.726 | 0.00-0.46 |
| RBC (10^12^/L) | 6.39 | 5.83 | 6.00 | 5.78 | 6.02 | | 5.62 | 6.41 | 5.94 | 0.32 | 0.343 | 0.998 | 0.611 | 5.50-9.00 |
| HGB (g/L) | 105.13^a^ | 88.63^b^ | 100.75^ab^ | 97.00^ab^ | 101.25^ab^ | | 99.50^ab^ | 106.88^a^ | 103.00^ab^ | 3.59 | 0.220 | 0.192 | 0.535 | 100.00-160.00 |
| HCT % | 33.41 | 30.37 | 31.28 | 30.03 | 32.48 | | 31.65 | 33.71 | 32.00 | 1.60 | 0.514 | 0.302 | 0.728 | 33.0-52.0 |
| MCV (fL) | 52.29 | 52.25 | 52.29 | 52.03 | 54.04 | | 53.54 | 52.61 | 53.89 | 0.98 | 0.904 | 0.065 | 0.857 | 51.0-73.0 |
| MCH (pg) | 16.44 | 17.05 | 16.86 | 16.86 | 16.90 | | 17.71 | 16.69 | 17.40 | 0.38 | 0.237 | 0.177 | 0.701 | 14.0-22.0 |
| MCHC (g/L) | 314.63^de^ | 326.62^ab^ | 322.37^bcd^ | 324.63^abc^ | 312.00^e^ | | 331.00^a^ | 317.00^cde^ | 323.00^bc^ | 2.74 | < 0.001 | 0.502 | 0.350 | 300.00-360.00 |
| PLT (10^9^/L） | 529.88^a^ | 408.67^ab^ | 399.13^b^ | 469.85^ab^ | 462.29^ab^ | | 398.50^b^ | 432.00^ab^ | 443.85^ab^ | 43.66 | 0.147 | 0.570 | 0.693 | 200.00-1000.00 |
| MPV (fL) | 9.33 | 9.46 | 9.68 | 9.44 | 9.43 | | 9.19 | 9.71 | 9.60 | 0.23 | 0.384 | 0.970 | 0.784 | 7.2-13.1 |
| PDW | 15.01^b^ | 15.33^ab^ | 15.26^b^ | 15.20^b^ | 15.26^b^ | | 15.74^a^ | 15.10^b^ | 15.34^ab^ | 0.16 | 0.077 | 0.166 | 0.340 | 12.0-17.5 |
| PCT % | 0.49^a^ | 0.32^b^ | 0.38^ab^ | 0.37^ab^ | 0.41^ab^ | | 0.29^b^ | 0.42^ab^ | 0.39^ab^ | 0.047 | 0.025 | 0.717 | 0.630 | 0.12-0.93 |

Abbreviation: WBC, white blood cell number; NEU, neutrophil number; LYM, lymphocyte number; MON, monocyte number; EOS, eosinophils number; BAS, basophils number; RBC, red blood cell number; HGB, hemoglobin; HCT, hematocrit; MCV, mean erythrocyte volume; MCH, mean erythrocyte hemoglobin content; MCHC, mean red blood cell hemoglobin concentration; PLT, platelet count; MPV, mean platelet volume; PDW, platelet distribution width; PCT, platelet hematocrit.

^1^ Data were the mean of 16 replicates per treatment.

^2^ Dietary treatment: CON, the control group, fed with the basal diet; HM1, the herbal mixture group 1, fed with the basal diet supplemented with 0.5 g herbal mixture/kg diet; HM2, the herbal mixture group 2, fed with the basal diet supplemented with 1.0 g herbal mixture/kg diet; HM3, the herbal mixture group 3, fed with the basal diet supplemented with 1.5 g herbal mixture/kg diet.

^3^ SEM = Standard error of the mean.

^a - c^ Value in the same row not sharing a common superscript mean significant difference (*p* < 0.05).
