# Supplementary material for: Effects of dietary supplement with a Chinese herbal mixture on growth performance, antioxidant capacity, and gut microbiota in weaned pigs
Source: Front Vet Sci. 2022 Aug 22;9:971647. doi: 10.3389/fvets.2022.971647 (PMC9442064; doi:10.3389/fvets.2022.971647)
Supplement: Supplementary file 7 [file Image_4.PDF]

Supplementary Material

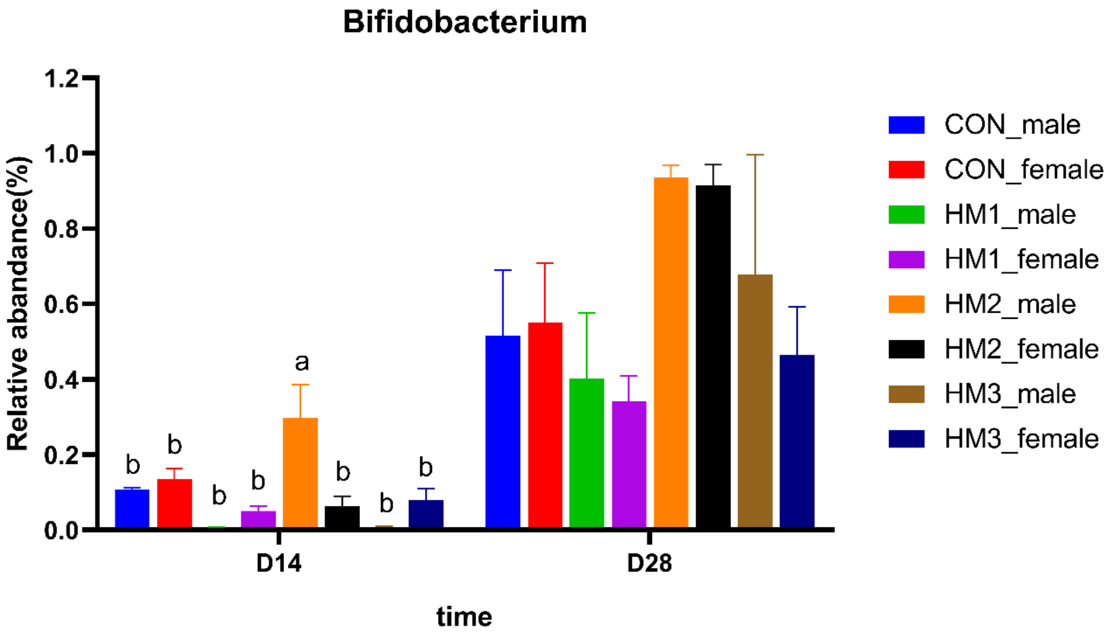

Supplementary Figure 4. Effect of treatment × gender interaction on the relative abundance of dominant genus of the intestinal microbiota.
